# Soft, stretchable, epidermal sensor with integrated electronics and photochemistry for measuring personal UV exposures

**DOI:** 10.1371/journal.pone.0190233

**Published:** 2018-01-02

**Authors:** Yunzhou Shi, Megan Manco, Dominique Moyal, Gil Huppert, Hitoshi Araki, Anthony Banks, Hemant Joshi, Richard McKenzie, Alex Seewald, Guy Griffin, Ellora Sen-Gupta, Donald Wright, Philippe Bastien, Florent Valceschini, Sophie Seité, John A. Wright, Roozbeh Ghaffari, John Rogers, Guive Balooch, Rafal M. Pielak

**Affiliations:** 1 L’Oréal Tech Incubator–California Research Center, San Francisco, California, United States of America; 2 L’Oréal Early Clinical, Clark, NJ, United States of America; 3 La Roche-Posay Dermatological Laboratories, Asnières Cedex, France; 4 MC10 Inc., Lexington, Massachusetts, United States of America; 5 Science and Engineering, and Frederick Seitz Materials Research Laboratory, University of Illinois at Urbana-Champaign, Urbana, Illinois, United States of America; 6 L’Oréal Tech Incubator, Clark, NJ, United States of America; 7 National Institute of Water and Atmospheric Research (NIWA), Lauder, Central Otago, New Zealand; 8 MyStudioFactory, The Landing, Media City UK, Salford, United Kingdom; 9 L’Oréal Research and Innovation, Aulnay sous Bois, France; 10 L’Oréal Tech Incubator, Clichy, France; University of Alabama at Birmingham, UNITED STATES

## Abstract

Excessive ultraviolet (UV) radiation induces acute and chronic effects on the skin, eye and immune system. Personalized monitoring of UV radiation is thus paramount to measure the extent of personal sun exposure, which could vary with environment, lifestyle, and sunscreen use. Here, we demonstrate an ultralow modulus, stretchable, skin-mounted UV patch that measures personal UV doses. The patch contains functional layers of ultrathin stretchable electronics and a photosensitive patterned dye that reacts to UV radiation. Color changes in the photosensitive dyes correspond to UV radiation intensity and are analyzed with a smartphone camera. A software application has feature recognition, lighting condition correction, and quantification algorithms that detect and quantify changes in color. These color changes are then correlated with corresponding shifts in UV dose, and compared to existing UV dose risk levels. The soft mechanics of the UV patch allow for multi-day wear in the presence of sunscreen and water. Two evaluation studies serve to demonstrate the utility of the UV patch during daily activities with and without sunscreen application.

## Introduction

UV radiation is essential for production of vitamin D and beneficial for human health, but over-exposure to UV has many associated risk factors, including skin cancer and photo-aging [1, 2]. The acute effects of excessive UVA and UVB exposure are usually short-lived and reversible. Such effects include erythema, pigment darkening and sunburn [3, 4]. Prolonged exposures even to sub-erythemal UV doses result in epidermal thickening and degradation of keratinocytes, elastin, collagen and blood vessels, thus leading to premature skin aging [5–7]. Clinical symptoms usually include increased wrinkling and loss of elasticity [8]. Studies have also shown that both UVA and UVB radiation have local and systemic immunosuppressive properties, which are believed to be an important contributor to skin cancer development [9, 10]. UV-induced DNA damage is an important factor in developing all types of skin cancer including melanoma, non-melanoma skin cancers, basal cell carcinoma and squamous cell carcinoma [11]. UVB is also known to induce changes in skin neuroendocrine functions [12–14] and affects global homeostasis [15–17]. Both UVA and UVB are strongly scattered by air, aerosols, and clouds. For high sun angles, when UV intensity is at the highest, cloud effects are similar at UVA and UVB wavelengths; however, for low sun conditions, the UVB attenuation tends to be stronger. Unlike UVB, UVA penetrates glass windows and therefore may result in excessive UV exposures even in an indoor environment [18]. In addition, UVA readily passes through the ozone layer resulting in higher intensities of the UVA portion of the solar spectrum at the earth surface. Continuous sunscreen protection and monitoring of personal UV exposures is therefore critical for better skin protection and prevention of skin cancer [19, 20].

Conventional wearable devices are rigid, bulky, and not compatible with sunscreens [21–23]. Recent development in material and power management enables integrated sensor system in more compact form [24]. Here we report on the design and development of a wearable, ultra-thin, stretchable, and breathable UV sensor for accurate quantification of personal UV exposures and quantification of sunscreen protection. The ultra-thin UV patch structure and elastic properties allow for conformal contact with the skin and continuous wear for up to 5 days. The UV patch is emollient and sunscreen compatible, allowing for skin care product and sunscreen application. It contains dyes that change color upon exposure to UV radiation. This color change is then quantified using a smartphone and a quantification algorithm. The algorithm uses a system of reference colors to allow for accurate quantification of the UV dye color change under different lighting conditions. In order to determine personal UV exposure levels and provide accurate personalized recommendations, the algorithm takes into account many parameters. First, the color change is converted to UVA radiation based on predetermined calibration tables that link color change to the amount of UVA radiation. Second, the corresponding UVB exposure is calculated using a pre-computed lookup table that gives the conversion factor as a function of the column amount of ozone in the atmosphere and solar zenith angle (SZA). GPS location of the user is determined and based on the user location and time SZA is calculated. Longitude, latitude and time are also used to extract the forecast ozone amount from satellite-measurements.

We tested the sensor in two evaluation studies. The first study demonstrated device functionality in different real life activities including swimming in the ocean, beach activities, showering, as well as compatibility with sunscreen and skin care product applications. It also helped us to further optimize and calibrate the device for accurate UV dose measurements. The second study demonstrated UV readout accuracy from the UV patch during controlled and real life daily activities.

## Experimental results

### Patch design

**Fig 1:**
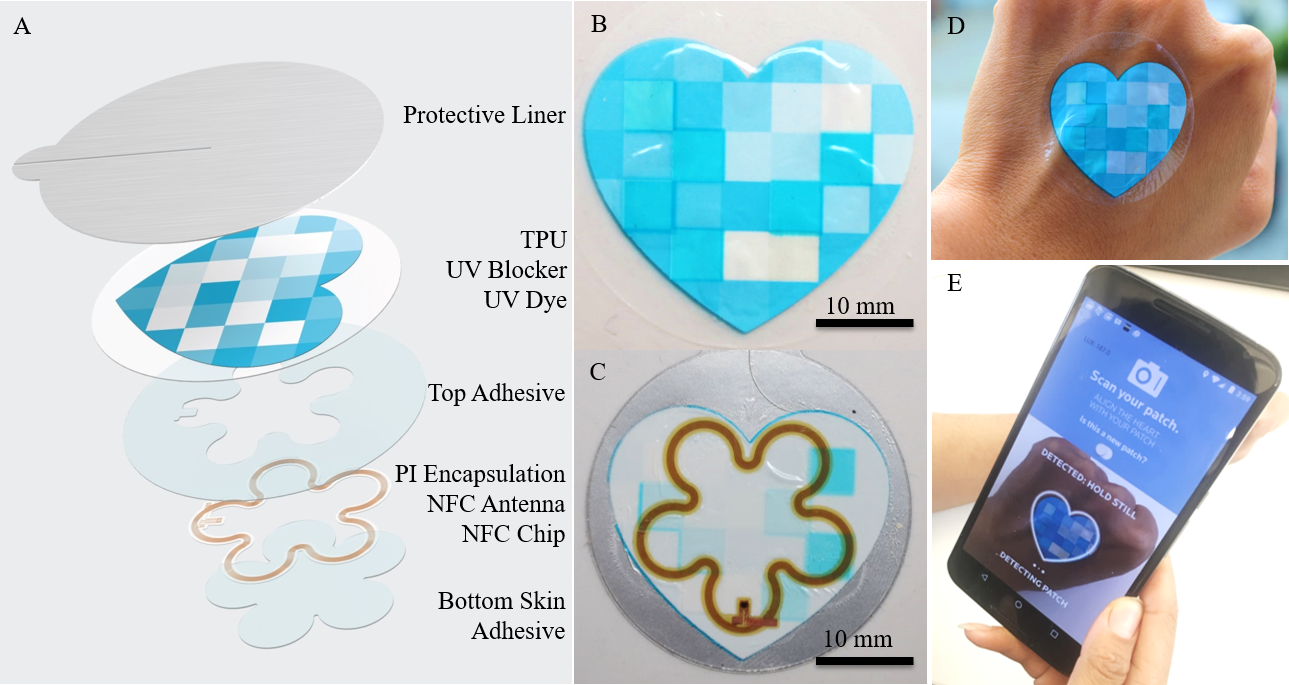
UV sensor structure. (A) Construction of the UV sensor (from the top to the bottom): protective liner with adhesive, permeable polyurethane (TPU, 16 μm) with printed UV ink, UV blockers and reference colors, top skin adhesive layer (25 μm), NFC antenna (yellow, 18 μm) and polyimide film encapsulation (PI, 12.7 μm), NFC antenna and chip (0.5 mm), polyethylene terephthalate layer (PET, 12 μm), bottom skin adhesive layer (25 μm), and bottom liner. (B) The front of the UV patch. (C) The back of the UV patch. Bar = 10 mm. (D) Wearing the UV patch on the back of one’s hand. (E) Reading the UV patch using the My UV Patch app.

The UV patch is designed to conform to the skin surface and provide a soft stretchable interface. When the UV patch is attached to the skin, it experiences similar UV exposure as the surrounding skin. An exposure to UV radiation results in patch color change, which is quantified using a smartphone (Fig 1). The UV sensing mechanism is composed of UV sensitive inks and blockers that are printed on a permeable polyurethane (TPU) film. The vapor transmission rate for the TPU is 18022±1404 g/m^2^/24hr. The tensile strength is 244 kg/cm^2^ in the direction of warp and 217 kg/m^2^ in the direction of weft; the 300% modulus is 165 kg/cm^2^ in the direction of warp and 174 kg/m^2^ in the direct of weft. The ultimate elongation rate is 369% in the direction of warp and 341% in the direction of weft. Below the TPU, the UV patch contains a Near Field Communication (NFC) chip and copper/polyimide (PI) antenna for communication with a smartphone. The NFC antenna is used to communicate with the smartphone and save unique user ID to identify patches in the software program. A thin layer of polyethylene terephthalate (PET) prevents the NFC and antenna from directly contacting the user’s skin. Below the PET layer, there is a thin layer of skin adhesive that couples the UV patch with the skin (Fig 1A).

### UV sensitive dyes design and optimization

**Fig 2:**
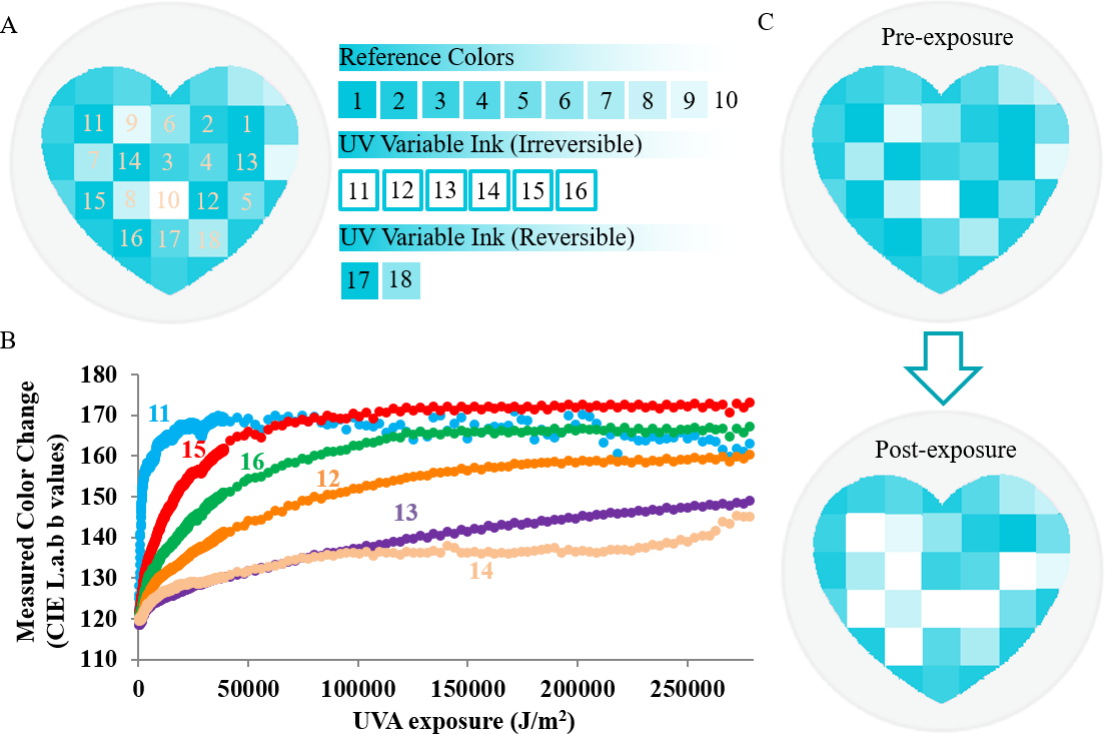
The mechanism of the UV sensor color change and color change quantification. (A) The UV patch is composed of a series of reference colors 1 to 10, UV variable ink squares 11 to 16, and UV reversible ink squares 17 and 18. The reference colors 1–10 correspond to the different colors of the UV ink squares when they are exposed to UV radiation. (B) The six UV sensitive ink squares change colors at distinctive rates when exposing to UVA radiation with square 11 being the most sensitive and square 14 being the least sensitive. The color change is quantified in CIE Lab color space. (C) Schematics showing the UV patch before and after exposure to UVA radiation.

When exposed to UV radiation the UV patch changes color, which is quantified by image processing algorithms (Fig 2). The UV patch is composed of ten reference color squares 1 to 10 and six irreversible UV sensitive ink squares 11 to 16 (Fig 2A). The six UV variable ink squares were optimized to change color at progressively decreasing rates in order to cover broad sensitivity range. This also allows us to average readouts from multiple squares for better data accuracy (Fig 2B). The ten reference colors are blue with 10 to 100% transparency by steps of 10%, respectively, with a minimum ΔE of 5 in between adjacent colors using the International Commission on Illumination (Commission Internationale de l'éclairage) (CIE)’s distance metric for colors. UV patches before and after UV exposure are shown in Fig 2C. An image of the UV patch is captured and processed by a cell phone application. The UVA dosage is then measured by quantifying the color change of the six UV variable ink squares using an algorithm within the cellphone application.

### Algorithm design

**Fig 3:**
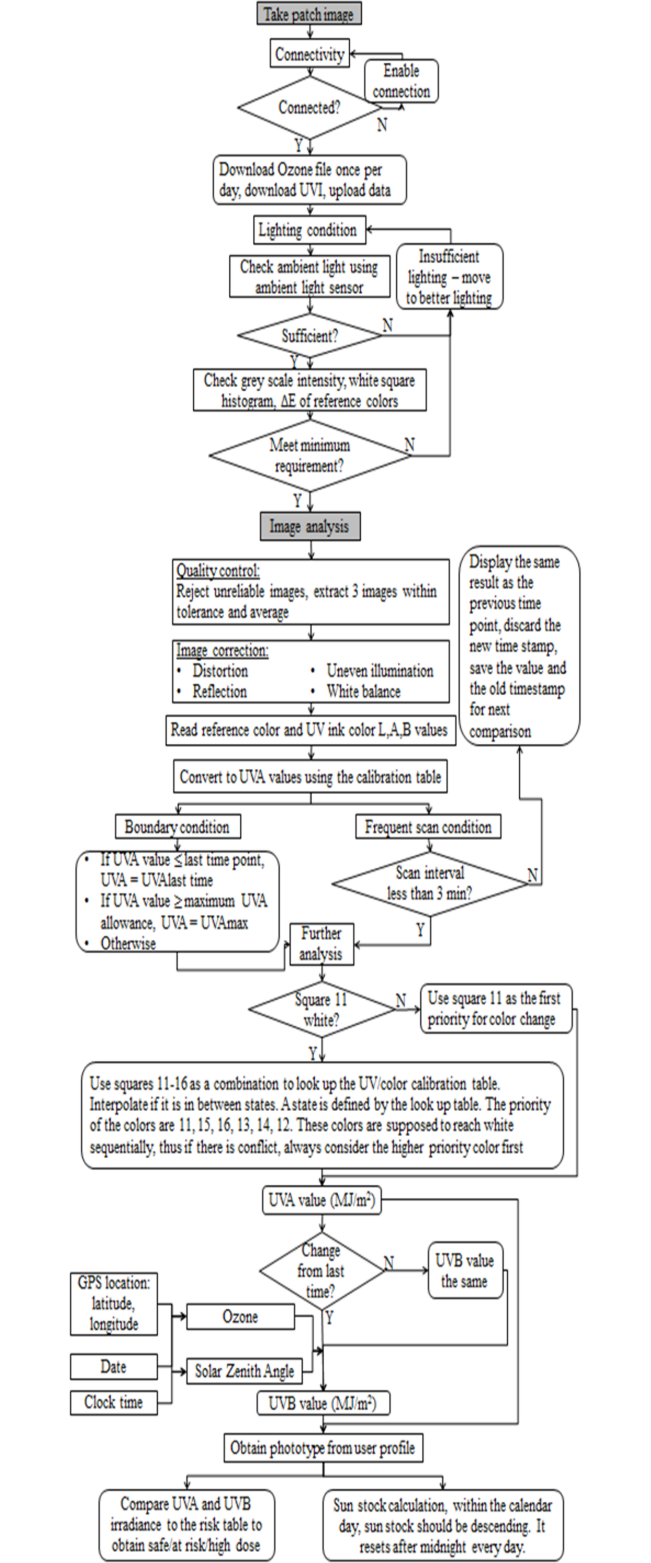
The app algorithm flowchart.

The application algorithm is designed to determine the user’s skin sensitivity to UV. The application also determines user’s location and the UV Index in the area. When the user scans the patch the application can calculates user’s personal UV doses and risk level and recommends sunscreen product that provides the best protection and comfort. The algorithm for the personal UV dose quantification include 4 subalgorithms: a) shape recognition and features location algorithm; b) lighting condition correction algorithm; c) color quantification algorithm; d) UV dose determination algorithm (Fig 3).

#### Shape recognition

The shape recognition algorithm is designed to automatically detect the patch shape and correct for any shape distortion. It then determines the location of all the UV sensitive squares and reference colors. Specifically, the first step is to determine whether a heart shape is present and its general position in the image, these are achieved by using Haar feature based cascade classifiers, which are trained using a large number of both positive images and negative images. The heart is then isolated from the image. The second step is to detect the shape more closely using feature matching, and further correct distortions using perspective control. Once the key points on the heart shape have been detected, the reference color squares and UV sensitive ink squares are then located using the template.

#### Lighting correction

The application takes multiple scans of the patch and every scan passes through a quality control process, which includes elimination of scans with uneven illumination and uneven light reflection. The images are then color corrected and white balance corrected. Only the best quality images are accepted and used for color quantification. Specifically, the colors are sampled from each reference color square and all UV sensitive ink squares. During the color sampling, the color histogram for each square is calculated and the center 50% of the pixel colors remain for further processing. This step is to remove wrinkles, light reflection and shadows resulting in reduced noise in the image. The sampled colors from each reference color squares are then compared to the “true color”, which is pre-determined by the color code of the inks. The color correction is performed for each square and the same correction matrix is applied to its surrounding UV sensitive ink squares.

#### Color quantification

After the images are corrected for lighting condition, the algorithm takes measurements of the color of the UV sensitive dyes and compares them to the reference colors. The reference colors are closely matched to the color of the UV sensitive dyes at different UV exposure levels. This allows for accurate color quantification at different lighting conditions, since any particular lighting condition affects the reference colors and UV sensitive dye colors to similar extent.

The image is processed in the (CIE) Lab color space (L*a*b space). The ΔE between squares 11–16 (UV variable ink squares) and squares 1–10 (reference color squares) are calculated, respectively, using Eq 1, where i = 11, 12, 13, 14, 15, 16 denotes the UV variable ink squares; j = 1, 2, 3, 4, 5, 6, 7, 8, 9, 10 denotes reference color squares. The conversion between the color and UVA values is through a look up table created during calibration and calculated using Eq 2, where i = 11, 12, 13, 14, 15, 16; j = 1, 2, 3, 4, 5, 6, 7, 8, 9, 10. Specifically, the UV variable ink square is matched to the closest reference color square by comparing ΔE. The UVA is interpolated between the UVA values that correspond to the two closest reference colors (Eq 2).

$${\Delta E}_{i,j}=\sqrt{{\left(L_{i}-L_{j}\right)}^{2}+{\left(a_{i}-a_{j}\right)}^{2}+{\left(b_{i}-b_{j}\right)}^{2}},$$ (1)

$$\left\{ \begin{array}{c} if\;{\Delta E}_{i,1}=\min_{j=1,\ldots ,10}{\Delta E}_{i,j}\;and\;\Delta E_{i,1}<threshold,\;{{UVA}_{i}=UVA}_{1},\\ Else,{UVA}_{i}=\frac{{{\Delta E}_{i,j}}^{-1}{UVA}_{j}+{{\Delta E}_{i,j-1+1}}^{-1}{UVA}_{j-1}}{{{\Delta E}_{i,j}}^{-1}+{{\Delta E}_{i,j-+1}}^{-1}}, \end{array}\right.,$$ (2)

The boundary condition and minimal scanning frequency are set as Fig 3 boundary condition and frequent scan condition. These are to further remove the noise of the readings. The source code can be found in S1 File.

#### UV dose determination

In order to determine user’s personal UV exposure levels and provide accurate recommendations, the algorithm takes into account many parameters. First, the color change is directly converted to UVA radiation dose based on predetermined calibration tables that link color change to UVA radiation. Second, the corresponding UVB exposure is calculated using a pre-computed lookup table that gives the conversion factor as a function of the column amount of ozone in the atmosphere and solar zenith angle (SZA) as previously described [25]. The lookup table was generated using the tropospheric ultraviolet and visible (TUV) radiative transfer model [26]. SZA is determined based on GPS location and time. The user latitude, longitude, and time are also used to extract the forecast ozone amount from satellite-measurements. In this conversion, the effects of clouds and aerosols are assumed to be similar at UVA and UVB wavelengths. It should be noted however, that for some organic aerosols (which are ubiquitous in densely populated areas) aerosol extinctions will generally be larger in the UVB region than in the UVA region. Thus the predicted UVB using this method will represent an upper limit. Ozone column amount data (measured in Dobson Units, DU, where 1 DU = 2.69 x10^16^ molecules per square centimetre), are extracted from daily global fields of ozone at the National Centers for Environmental Prediction at National Oceanic and Atmospheric Administration (NCEP/NOAA). UVA and UVB results are then cross-checked with the maximal values expected for the user location determined based on UVI forecasting webservices. Again, precomputed lookup tables, which are functions of ozone and SZA, are used to relate the quantities. This process prevents sporadic and erroneous readouts. If an Internet connection is not available, the result is cross-checked with lookup tables that relate maximal UVI data with corresponding maximal values for UVA and UVB at different geographical locations and time.

Note that according to the CIE and German Industrial Standard (DIN 5031), the wavelength threshold between UVB and UVA is 315 nm [25].

### Personal daily safe UV doses and risk levels

The personal daily safe UV doses are calculated based on the skin phototype and minimal erythema dose (MED) (Table 1). The skin phototype is determined according to the Fitzpatrick phototype scale, on a simplified user questionnaire completed by the user when the user first opens the app. The maximal daily safe UV dose is set to 0.4 MED for each skin phototype and it is based on studies demonstrating that some degree of UV exposure induces skin damage can be observed after exposure to 0.5 MED [19, 20] or even lower-level summer sunlight [7]. The rate of change of the UV exposure throughout the day is defined as “exposure” and it is calculated for every scan for the time between the current and previous patch scan. It is divided into 3 zones: 1) Green–on track to stay within the daily safe UV dose; 2) Orange–at risk to exceed the daily safe UV dose; 3) Red–high risk of UV overexposure (Table 2).

**Table 1:** Personal UV daily sunstock.

Skin Phototype	UV Daily Sunstock (MJ/m^2^)
**1**	0.015
**2**	0.022
**3**	0.029
**4**	0.037
**5**	0.044
**6**	0.044

**Table 2:** Personal UV risk determination.

Skin Phototype	UV Risk (MJ/m^2^/hr)		
	Safe Green Zone	At Risk Orange Zone	Too High Red Zone
**1**	**<0.0015**	**0.015–0.003**	**≥0.003**
**2**	**<0.0022**	**0.0022–0.0044**	**≥0.0044**
**3**	**<0.0029**	**0.0029–0.0058**	**≥0.0058**
**4**	**<0.0037**	**0.0037–0.0074**	**≥0.0074**
**5**	**<0.0044**	**0.0044–0.0088**	**≥0.0088**
**6**	**<0.0044**	**0.0044–0.0088**	**≥0.0088**

### Sensor validation

**Fig 4:**
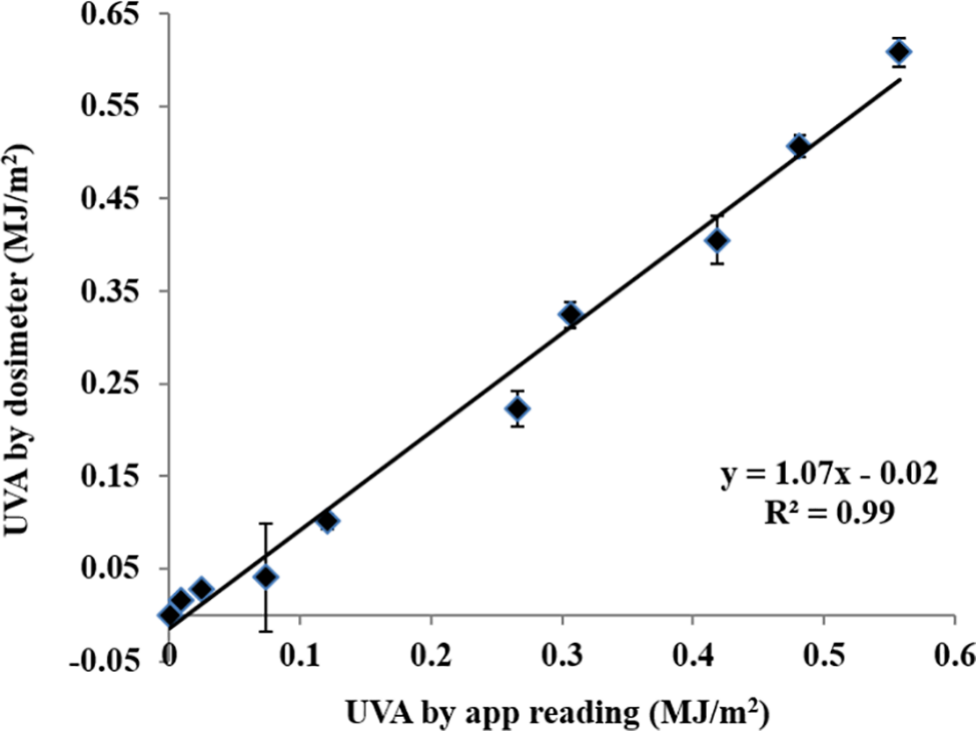
Comparison of the UV patch and Scienterra UV dosimeter. 10 UV doses were examined, 3 replicates for each UV dose, *p*<0.00001.

We used electronic Scienterra UV dosimeters as reference devices for the UV patch calibration. The Scienterra dosimeters were calibrated at the Solar Irradiance Monitoring Station at the National Renewable Energy Laboratory (NREL) (Davis, California, USA). The patch was then validated under natural sun light and under artificial light using an Advanced Beam Optics Design Class A+AA Solar Simulator Model TSS-156 with AM1.5G spectrum from 300 to 1800 nm (OAI Inc. USA). A strong correlation was demonstrated between the UV patch readings and the Scienterra UV dosimeter readings (R^2^ = 0.99, *p*<0.00001 at the range of 0 to 0.6 MJ/m^2^, 3 replicates; Fig 4).

**Fig 5:**
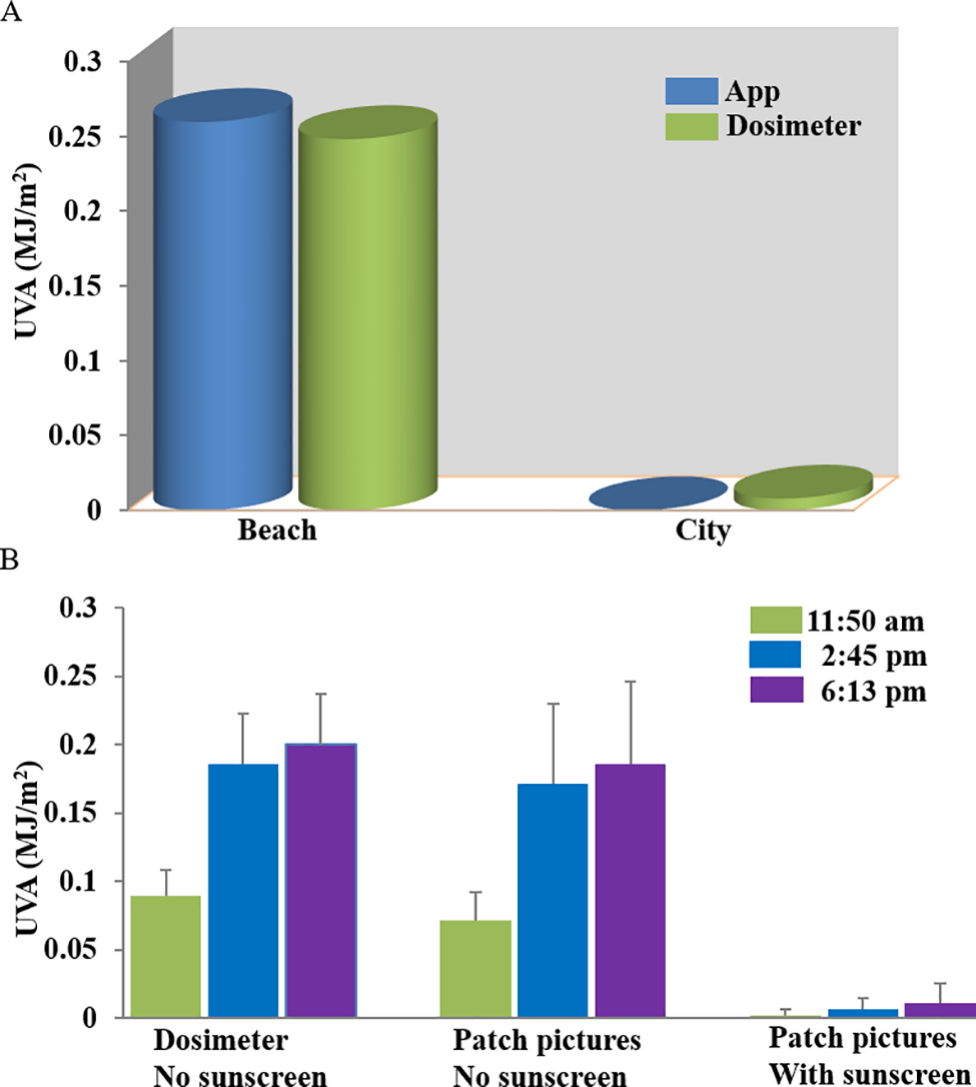
Clinical evaluation of the UV patch. (A) The study subjects wore the UV patches and Scienterra dosimeters during regular city and beach activities. Both devices showed agreement in UV dose measurements. (B) The study subjects conducted controlled activity: single file walk in specified directions. The activity was repeated in the morning, afternoon, and evening. Each study subject wore one Scienterra dosimeter and two UV patches: one without sunscreen and the other one with sunscreen applied on it. Both the electronic dosimeter and the UV patch without sunscreen showed consistent results. *p* < 0.0001, n = 24. The UV patch covered with sunscreen showed significant reduction in measured UV radiation.

**Fig 6:**
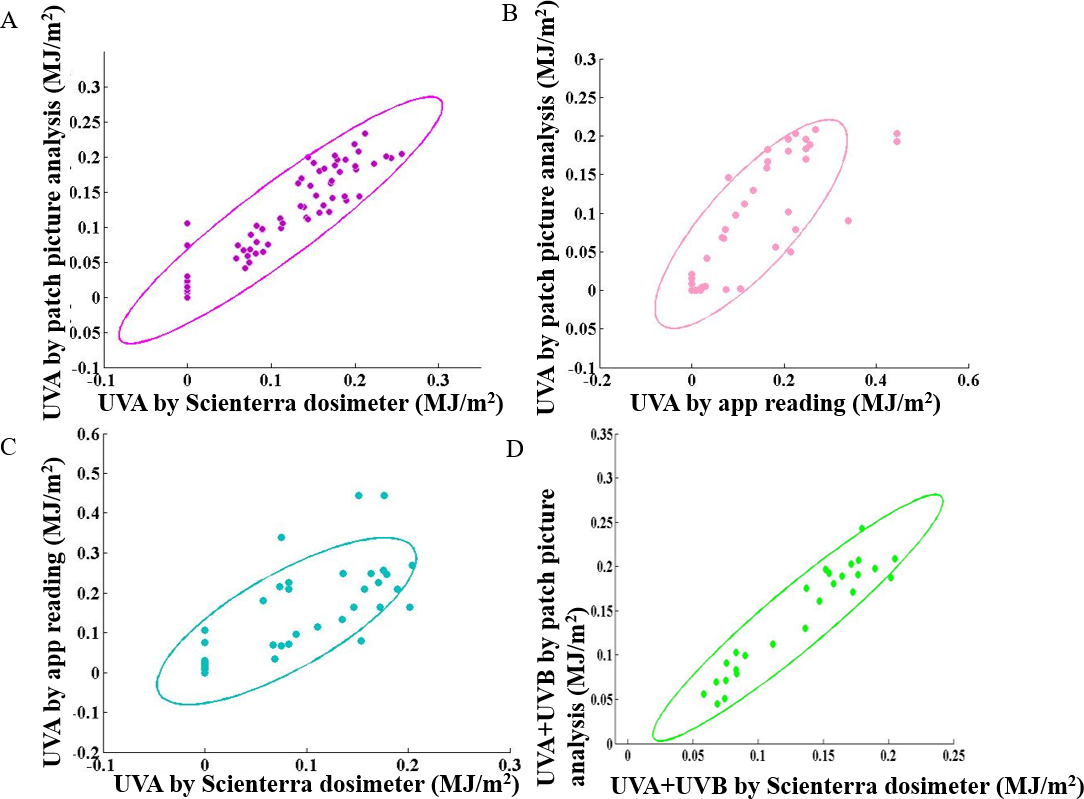
Comparison of UV readings among UV patch image analysis, Scienterra dosimeter and the mobile application. UVA readings by patch picture analysis showed high correlation with Scienterra dosimeter readings, which validates the UV sensor image technique *p* < 0.0001, *r* = 0.88, n = 30 (A). When compared between the patch picture analysis and app reading, the data still shows good correlation but the fast patch scanning requirement for improved user experience affected data quality *p* < 0.0001, *r* = 0.92, n = 30 (B). Similar result is shown between Scienterra dosimeter and app reading *p* < 0.0001, *r* = 0.92, n = 24 (C). The total UV dose shows a good correlation between the Scienterra dosimeter and patch picture analysis *p* < 0.0001, *r* = 0.87, n = 24 (D). The 95% prediction ellipse is shown. The strong correlation among the three measurements further validates the sensor system.

The UV patch was then evaluated on human volunteers in the clinical study with approval granted by The Quebec International Review Board, Ontario Canada. The study was designed to test the patch UV readout accuracy during controlled and real life daily activities. The subjects received an average of 0.2593±0.0499 MJ/m^2^ UVA exposure from 10 am to 2 pm during the free beach activity, and 0.0000±0.0000 MJ/m^2^ UVA exposure from 3 pm to 4 pm during the free city walk measured by the UV sensor patch app reading. The Scienterra dosimeters read similar values for the beach activity but higher values for city walk, 0.2479±0.0248 MJ/m^2^ for the beach activity and 0.0078±0.0048 MJ/m^2^ for the city walk, respectively (Fig 5A, S1 Table). The UV sensor patch is compatible with sunscreen. Measured by the UV sensor patch, the sunscreen greatly reduced the UV exposure during an intermittent UV exposure in the morning, afternoon and evening. Without the sunscreen protection, the UVA exposure was 0.0711±0.0215 MJ/m^2^, 0.1716±0.0581 MJ/m^2^, 0.1861±0.0600 MJ/m^2^ measured at 11:50 am, 2:45 pm and 6:13 pm, respectively. With the sunscreen protection, the UVA exposure was 0.0021±0.0047 MJ/m^2^, 0.0061±0.0084 MJ/m^2^ and 0.0111±0.0139 MJ/m^2^, respectively (Fig 5B, S2 Table). We compared the Scienterra UV dosimeter readings to the UV patch image analysis results, similar UVA exposure was shown without the sunscreen protection, 0.0896±0.0185 MJ/m^2^, 0.1858±0.0372 MJ/m^2^ and 0.2001±0.372 MJ/m^2^, at 11:50 am, 2:45 pm and 6:13 pm, respectively. Due to the limitation of Scienterra dosimeters, the sunscreen effect could not be measured (Fig 5B, S3 Table). Patch images, UV dosimeter readings and app readings were then compared. The statistical analysis shows strong correlation between the UVA levels measured by Scienterra dosimeters and the UV patch picture analysis (*p* < 0.0001, *r* = 0.88, n = 30) (Fig 6A), between the UV sensor patch app reading and the patch picture analysis (*p* < 0.0001, *r* = 0.92, n = 26) (Fig 6B), as well as between the Scienterra dosimeter and the UVA sensor patch app reading (*p* < 0.0001, *r* = 0.92, n = 26) (Fig 6C). The raw data are shown in S2 Table. The total UV dose which includes both UVA and UVB measured by Scienterra dosimeter and patch picture analysis is highly correlated, (*p* < 0.0001, *r* = 0.87, n = 26) (Fig 6D). The 95% prediction ellipse is shown. The strong correlation among the three measurements further validates the sensor system. The discrepancy between the UV sensor patch app reading and the other two measurements are due to the fast patch scanning requirement for improved user experience.

### Personal UV data collection from different geographical location

**Fig 7:**
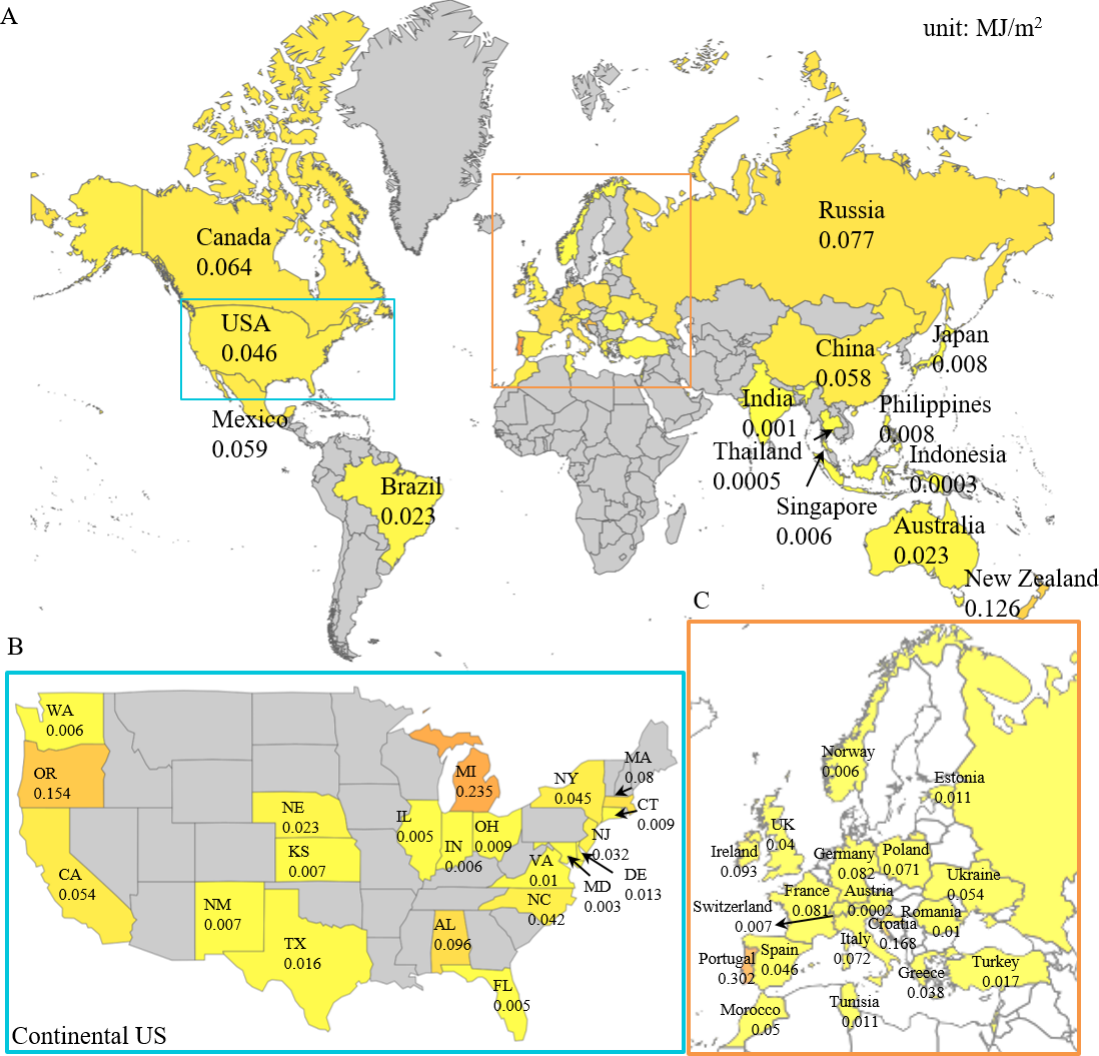
World average UV exposure. (A) A world average UV exposure is generated based on the My UV Patch app user data from June 6^th^, 2016 to August 18^th^, 2016. Zoom in maps are shown for continental US (B) and part of Europe (C). The country and state that contributed the data are labeled in yellow to red, color map is generated by normalizing the UV exposures to range between 0 (minimum UV exposure, yellow) and 1 (maximum UV exposure, red).

The use of cell phone app enables data visualization on a large scale. Half a million patches were distributed at no charge in 23 countries through La Roche-Posay. Anonymized usage data was collected on a cloud server and analyzed. Fig 7 shows average personal UV exposure levels based on the My UV Patch app user data (Fig 7). Maximum UV exposure for each cell phone device is collected and averaged at country level worldwide (Fig 7A), state level in US (Fig 7B) and country level in Europe (Fig 7C). Data from a total of 39 countries and 26 US states were received between June 6, 2016 and August 18, 2016, and were processed for the map. The additional number of country is possibly due to user travel.

## Discussion

The Ultraviolet Index (UVI) is a commonly used international standard UV measurement scale [27, 28]. UVI represents the strength of sunburn-producing UV radiation. It is a scaled version of the erythemally weighted irradiance falling on a horizontal surface; therefore, it implicitly includes a zenith angle cosine dependence. For complex surface topographies such as human skin, the personal exposure can be quite different from the idealized case of the radiation on a horizontal surface. Depending on the geometry of solar position and surface orientation, personal UV exposure can either be greater or less than the exposure predicted from UVI, sometimes by factors larger than 30%. For example, under cloudy conditions, the real UV exposure can be less than 50% of UVI [29, 30]. The UV dose received by human skin depends also on body-site. For example, UV exposure on the thigh will generally be less than on the top of the head or shoulder. However, it has been shown that the radiation on some specific sites, such as the wrist, can be considered as representative of a mean value [31].

Another limitation of UVI is that it is heavily weighted toward UVB, and there is an increasing body of data indicating that UVA also contributes to skin aging and skin cancer [6, 8, 29, 32]. Moreover, unlike UVB, UVA radiation can penetrate through glass windows. The UVA portion of the solar spectrum also has a much higher intensity than UVB (partly due to attenuation of UVB by atmospheric ozone, UVA doses are typically 20 or 30 times greater than UVB doses). These factors result in human skin being exposed to higher cumulative UVA doses than UVB doses. Because UVA does not contribute to suntan or sunburn as much as UVB, people are often not aware of excessive UVA exposures, especially on cloudy days or in indoor environments. In addition, skin damage from UV exposure is not immediately apparent. The erythemal reaction can occur more than 12 hours after exposure [33] making it difficult for an average person to know what is the safe amount of UV dose. Thus, tailoring personalized UV exposure advice is important, as also supported by previous studies [7].

Modern broadband sunscreens provide effective protection against UVA as well as UVB radiation; however, even with sunscreen protection the skin can still be exposed to damaging UV doses. Therefore, continuous personal UV exposure monitoring in the presence and absence of sunscreen is critical for better skin protection.

The objective of this project was to design and develop a low cost, wearable UV sensor for accurate quantification of personal UV exposures and degree of protection by sunscreens. My UV Patch provides continuous personal UV exposure monitoring with or without sunscreen applied and provides the user with recommendations for better UV protection. It is stretchable, breathable, and has similar mechanical properties to human skin. The user can apply sunscreen on the patch the same way as it is applied on the rest of the body. The patch then helps to provide information on how much the sunscreen reduced the user’s UV exposure. We conducted two clinical studies that demonstrated sensor use versatility and data accuracy. The patch maintained accurate readouts even after exposure to ocean water, high heat and humidity, excessive sweat, skin care products and sunscreens. In fact, a main advantage of the patch is that it is capable of measuring UV doses in the presence of sunscreen, therefore providing direct measurement of the user’s UV exposure when protected with sunscreen. We evaluated patch readout accuracy against Scienterra electronic dosimeters, which have been widely used in research studies involving personal UV exposures [34]. The patch colorimetric analysis showed good correlation to the Scienterra devices. The ultimate test was through the wide distribution of the device to the public in July 2016, and the analysis of the resulting data. Half a million of patches were distributed in 23 countries around the world at no charge through La Roche-Posay. This allowed us to acquire data on daily personal UV doses in different geographical locations and relate them to sunscreen usage and UVI in these locations (Fig 7).

## Methods

### Printing

The reference colors are printed on TPU films (DingZing Advanced Materials Inc., Taiwan) using roller printing. The UV ink and blockers (Spectra Group Inc., USA) are then printed using screen printing with mesh size ranging from 110 to 380 um. Below the TPU film is the near field communication antenna (NXP semiconductors). The adhesives used in the patch are medical grade (Flexcon Inc., USA).

### Calibration methods

To calibrate the response of the UV dyes, the UV sensor patches are first calibrated under natural sun light with solar UV radiation. The solar UV radiation is measured by electronic UV dosimeters (Scienterra Inc, New Zealand). The Scienterra dosimeters are pre-calibrated against the instruments at the solar irradiance monitoring station in the UV-B monitoring and research program by NREL. The Scienterra dosimeters are also compared with radiative transfer calculations using TUV radiation model on several clear days in San Francisco.

During the development process, large batches of UV sensor patches are exposed to an Advanced Beam Optics Design Class A+AA Solar Simulator Model TSS-156 with AM1.5G spectrum from 300 to 1800 nm (OAI Inc. USA). The UV intensity is measured using the OAI 308 Meter and a 365 nm probe (OAI Inc. USA) and is kept constant. The images of the UV sensor patch are captured by a Nikon D5100 digital camera (Nikon Inc, USA). Images are processed using a Matlab routine (Mathworks Inc., USA). The response curves of the UV sensor patch are compared between the solar simulator exposure and natural sun light exposure to achieve consistency.

### Algorithm and software

The image processing algorithm was written in Matlab then implemented using C/C++ with the OpenCV library for both Android and iOS apps. Part of the image processing is written in Objective-C for iOS and Java for Android. The visualizing of the world UV map is achieved by a custom web framework built in house using JavaScript, Node.js, require.js, HMTL and CSS.

An Android and iOS version of the software can be downloaded from Google Play and App Store under the name “My UV Patch”.

### Clinical study protocol

Healthy volunteers with skin phototype IV—VI according to the Fitzpatrick classification [35], with intact, healthy skin in the investigational areas were screened and recruited by Hill Top Research, TX, USA. A total number of 11 volunteers between 18 and 45 years old were enrolled into the study with 5 males and 6 females, among whom, 6 were Fitzpatrick skin phototype IV, 1 was phototype V and 4 were phototype IV. The average age of the volunteers was 27.4 ± 8.7 (n = 11). On each day of the study, the investigational areas including inner forearm, wrist and back of hands were gently cleaned with isopropyl alcohol patches. Pictures of the investigational areas were taken before patch application and after patch removal to evaluate skin irritation. Each subject wore one patch on the back of their left hand, one on the back of right hand and one on the inner forearm, respectively. Each subject wore a UVA Scienterra dosimeter and a UVB Scienterra dosimeter on their wrist. The patches on the back of hands are kept for multi-day continuous measurements while the patch on inner forearm was replaced daily. The patch evaluation study was conducted in St. Petersburg, Florida. On day 1, subjects walked along the pre-set route in the morning, at noon and in the afternoon for four miles, respectively. On day 2, subjects conducted beach activity for two hours and followed by one hour free city walk following a pre-determined route. On day 3, subjects repeated day 1 activity with La Roche-Posay Anthelios 30 sunscreen (2 mg/cm^2^) applied on the skin as well on one of the UV sensor patches. Subjects took pictures of the patches with the smartphone camera. At the same time, patch pictures were also taken by a trained instructor. The trained study coordinators used an iPhone camera to acquire patch images at a fixed angle to avoid glare and shadow. Patch images, UV dosimeter readings and app readings were compared.

### Statistical analysis

Scatterplot matrices of the descriptors by time allow visualization of pairwise relationships. The associated Pearson correlation coefficients are displayed as tables or heat map representations. All statistical analyses have been performed using SAS statistical software release 9.3, SAS Institute Inc., Cary, NC, USA, and JMP statistical software release 10.0 (JMP is a trademark of SAS Institute).

### Ethics statement

The clinical evaluation studies were performed in Florida, USA, with approval granted by the Quebec International Review Board, Ontario Canada. The studies were conducted in compliance with the ethical principles in the Declaration of Helsinki, in accordance with the Good Clinical Practice guidelines (ICH Topic E6) and compliance with local regulatory requirements.

Prior to entry into the study, the consent for each subject participating was obtained. Subjects were provided an Informed Consent document written in national language in easily understood wording. The subject reviewed the document and was provided the opportunity to ask questions and receive clarification from study personnel. The subject and the Investigator had to date and sign the last page of the Informed Consent Form to confirm that all information regarding this study was provided and that consent has been obtained. Two original copies for each subject participating in the study were signed. One original was intended for the subject, another one for the Investigator File. The consent statement met the requirements of applicable regulation. The Investigator informed each subject as to the purpose and nature of the study in compliance with applicable regulations. The signed Informed Consent Form was obtained before engaging any study procedure with the subject.

The multi-country data was anonymous and the consent for each user was obtained from users who downloaded from the App Store and Google Play. Digital regulations on data collection were followed.

## Supporting information

S1 File The matlab routine UVreader.m to read the color from the UV patches. (PDF)

S1 Table The individual level data for the clinical study where subjects did free beach activities and city walk. (PDF)

S2 Table The individual level data for the clinical study where subjects walked along the pre-set route in the morning, at noon and in the afternoon for four miles, respectively, with La Roche-Posay Anthelios 30 sunscreen applied on the skin as well on one of the UV patches. (PDF)

S3 Table The individual level data for the clinical study where subjects walked along the pre-set route in the morning, at noon and in the afternoon for four miles, respectively. (PDF)
