# Supplementary material for: Soft, stretchable, epidermal sensor with integrated electronics and photochemistry for measuring personal UV exposures
Source: PLoS One. 2018 Jan 2;13(1):e0190233. doi: 10.1371/journal.pone.0190233 (PMC5749742; doi:10.1371/journal.pone.0190233)
Supplement: S1 File — (PDF) [file pone.0190233.s001.pdf]

S1 File: The matlab routine UVreader.m to read the color from the UV sensor patches.

```
%%This program read in the color values from all squares in the patch.
% all rights belong to L'Oreal USA.
% Program written to analyze UV patch images for research.
clear all;
clc;
close all;

%request user input for image file location
pathfolder = uigetdir('start path', 'pick a folder');
cd(pathfolder);

if ~isdir(pathfolder)
  errorMessage = sprintf('Error: The following folder does not exist:\n%s', pathfolder);
  uiwait(warndlg(errorMessage));
  return;
end

%make the table
numberofsquares = inputdlg({'How many squares to analyze?','How many reference
colors'},'Input number of squares');
n = str2num(numberofsquares{1});
refn = str2num(numberofsquares{2});
numOfelements = 4*n+4; %the first column is row, the next three is for reference color 10

answer = inputdlg({'Output file name', 'Date of data collection', 'Name of collector'}, 'Input the
file name to store data');
Outputfilename = strcat(answer(1),'_', answer(2), '_', answer(3),'.xlsx');

Rname = ['Row',' ', 'Lref10',' ','Aref10',' ','Bref10'];

for i= 1:n
    Rname = [Rname, ' ', sprintf('L%d A%d B%d deltaE%d', i,i,i,i)];
end
Rnames = strsplit(Rname,' ');
Rnames = Rnames(~cellfun('isempty', Rnames));  %remove empty cells
filePattern = fullfile(pathfolder, 'DSC_*.jpg'); %CHANGE FILE FORMAT HERE IF NEEDED
jpgFiles = dir(filePattern);
filenumber = length(jpgFiles);

T = array2table(zeros(filenumber,numOfelements));
T.Properties.VariableNames = Rnames;
%% choose the squares
% choose one image to locate the squares
```

```
imageFile = uigetfile('DSC_*.jpg','choose one image to locate the squares');
Im = imread(imageFile);
imshow(Im);

sqrlocation = zeros(n,4);  % square locations
location = zeros(n,4); %temporary storage
%choose a reference color #10
reflocation = zeros(1,4); %reference color location
ref = zeros(1,4); %temporary store reference color location

%select the reference color to calculate Delta E.
uiwait(msgbox('Please select reference color #10 to calculate Delta E'));

j = 1;
while j >0
    ref = getrect();
    choice = questdlg('Are you ok with reference color #10?','Confirmation', 'Yes, continue', 'No,
repick', 'Yes, continue');
    if strcmp(choice, 'Yes, continue') == 1
        reflocation = ref;
        j = 0;
    else
        j = 1;
    end
end

for i = 1:n
    uiwait(msgbox(sprintf('Please choose square #%d, hold your mouse and drag', i)));
    k = 1;
    while k > 0
        location(i,:) = getrect();
        choice = questdlg('Would you like to pick the next square?','Confirmation', 'Yes, continue',
'No, repick', 'Yes, continue');
        if strcmp(choice, 'Yes, continue') == 1
            sqrlocation(i,:) = location(i,:);
            k = 0;
        else
            k = 1;
        end
    end
end

close('all');

%%extract the LAB values of the squares
for k = 1:length(jpgFiles)
```

```
    baseFileName = jpgFiles(k).name;
    c = strsplit(baseFileName, {'.','_'},'CollapseDelimiters',true);
    Row = c(2);
    fullFileName = fullfile(pathfolder, baseFileName);
    fprintf(1, 'Now reading %s\n', fullFileName);
    imageArray = imread(fullFileName);
    WP = whitepoint('D65');
    cform = makecform('srgb2lab', 'AdaptedWhitePoint', WP);
    T.Row(k) = str2num(char(Row));

    %reference color #10 L,A,B
    crop = imcrop(imageArray, reflocation);
    lab_crop = applycform(crop, cform);
    Lref10 = mean2(lab_crop(:, :, 1));
    Aref10 = mean2(lab_crop(:, :, 2));
    Bref10 = mean2(lab_crop(:, :, 3));
    %put in the table
    T(k,2) = table(Lref10);
    T(k,3) = table(Aref10);
    T(k,4) = table(Bref10);

    for i = 1:n
        crop = imcrop(imageArray, sqrlocation(i,:));
        lab_crop = applycform(crop, cform);
        L = mean2(lab_crop(:, :, 1));
        A = mean2(lab_crop(:, :, 2));
        B = mean2(lab_crop(:, :, 3));
        deltaE = sqrt((L-Lref10)^2+(A-Aref10)^2+(B-Bref10)^2);
        j = 4*(i-1)+5;
        %put in the table
        T(k,j) = table(L);
        T(k,j+1) = table(A);
        T(k,j+2) = table(B);
        T(k,j+3) = table(deltaE);
    end
end

writetable(T,char(Outputfilename));
```
