# Supplementary material for: Soft, stretchable, epidermal sensor with integrated electronics and photochemistry for measuring personal UV exposures
Source: PLoS One. 2018 Jan 2;13(1):e0190233. doi: 10.1371/journal.pone.0190233 (PMC5749742; doi:10.1371/journal.pone.0190233)
Supplement: S1 Table — (PDF) [file pone.0190233.s002.pdf]

S1 Table: The individual level data for the clinical study where subjects did free beach activities and city walk (Unit: $MJ/m^2$).

| subject # | UVA by Scienterra Dosimeter | |
|---|---|---|
| | **Beach** | **City** |
| 1 | 0.333068399 | 0.000821602 |
| 2 | 0.191557971 | 0.000429231 |
| 3 | 0.275551797 | 0.001183352 |
| 4 | 0.149721718 | 0.000429231 |
| 5 | 0.440418035 | 0.001643191 |
| 6 | 0.187852329 | 0.031811819 |
| 7 | 0.187581669 | 0.000536112 |
| 8 | 0.209611048 | 0.00072263 |
| 9 | 0.250877226 | 0.046779711 |
| 10 | 0.227432754 | 0.000399126 |
| 11 | 0.27357097 | 0.000736653 |

| | UVA by app reading | |
|---|---|---|
| Device ID | Beach | City |
| 10E1E2F5-1DEA-BC94-30C1-0C390566900F | 0.5188 | 0 |
| 23EA04A3-A67A-31F6-2C25-A88A66F5DF98 | 0.165151 | 0 |
| 2FE31325-B255-626C-ED19-856F72676661 | 0.0699 | 0 |
| 3B0FDA29-6DED-B5C7-CA71-15F0677FC972 | 0.4298 | 0 |
| 8F59414F-5CCA-1CA2-7AAD-2CF946B455CE | 0.203033 | 0 |
| 45EE2A7A-3661-A02F-58BC-6C71D37A710B | 0.277354 | 0 |
| 0f607264fc6318a92b9e13c65db7cd3c | 0.2408936 | 0 |
| d455bc2aee4693cdde35496f9e9582f3 | 0.33741528 | 0 |
| 7e0a031deaa9184c299c2b1abd5d15ea | 0.092135966 | 0 |

| | Beach | | City | |
|---|---|---|---|---|
| | app reading | Scienterra Dosimeter | app reading | Scienterra dosimeter |
| average | 0.259386983 | 0.247931265 | 0 | 0.00777206 |
| SEM | 0.04986417 | 0.024806835 | 0 | 0.004807697 |

UV app data were de-identified, therefore, subjects are identified by device ID. Two subjects, subject 2 and 3, had malfunction app for their devices, thus their app data were not received.
