# Supplementary material for: Soft, stretchable, epidermal sensor with integrated electronics and photochemistry for measuring personal UV exposures
Source: PLoS One. 2018 Jan 2;13(1):e0190233. doi: 10.1371/journal.pone.0190233 (PMC5749742; doi:10.1371/journal.pone.0190233)
Supplement: S2 Table — (PDF) [file pone.0190233.s003.pdf]

S2 Table: The individual level data for the clinical study where subjects walked along the pre-set route in the morning, at noon and in the afternoon for four miles, respectively, with La Roche Posay Anthelios 30 sunscreen applied on the skin as well on one of the UV sensor patches (Unit: $MJ/m^2$).

| | cumulative: cumulative through the day | | | | | | | | |
|---|---|---|---|---|---|---|---|---|---|
| | day 3 walk - morning | | | day 3 walk until noon | | | day 3 walk until afternoon | | |
| | w/o Sunscreen | | w/ Sunscreen | w/o Sunscreen | | w/ Sunscreen | w/o Sunscreen | | w/ Sunscreen |
| Subject # | Scienterra dosimeter | Patch picture analysis | Patch picture analysis | Scienterra dosimeter | Patch picture analysis | Patch picture analysis | Scienterra dosimeter | Patch picture analysis | Patch picture analysis |
| 1 | 0.090175 | 0.0654 | 0.0003 | 0.189618 | 0.1388 | 0.021 | 0.204911 | 0.1448 | 0.0289 |
| 2 | 0.089804 | 0.0974 | 0.0003 | 0.19944 | 0.2192 | 0.0004 | 0.21227 | 0.2344 | 0.0004 |
| 3 | 0.112165 | 0.0984 | 0.0004 | 0.223332 | 0.1914 | 0.0006 | 0.237541 | 0.2012 | 0.0034 |
| 4 | 0.059898 | 0.0741 | 0.0002 | 0.131912 | 0.1628 | 0.0004 | 0.144345 | 0.2002 | 0.0004 |
| 5 | 0.113795 | 0.1054 | 0.0011 | 0.24294 | 0.1989 | 0.0011 | 0.255534 | 0.2049 | 0.0011 |
| 7 | 0.096458 | 0.0754 | 0.0003 | 0.182009 | 0.1789 | 0.0004 | 0.200532 | 0.1874 | 0.0371 |
| 10 | 0.08203 | 0.0627 | 0.0027 | 0.173108 | 0.1415 | 0.0103 | 0.187922 | 0.1441 | 0.0114 |
| 11 | 0.072117 | 0.0595 | 0.0003 | 0.144307 | 0.1116 | 0.0004 | 0.157731 | 0.121 | 0.0004 |

| | cumulative: cumulative through the day | |
|---|---|---|
| | w/ Sunscreen | |
| Record # | App reading | Patch picture analysis |
| 1 | 0 | 0.0003 |
| 2 | 0.019863 | 0.0003 |
| 3 | 0 | 0.0004 |
| 4 | 0.0745 | 0.0011 |
| 5 | 0.022998 | 0.004575 |
| 6 | 0.006878 | 0.0003 |
| 7 | 0 | 0.021 |
| 8 | 0 | 0.0004 |
| 9 | 0 | 0.0155 |
| 10 | 0.029713 | 0.00535 |
| 11 | 0.010167 | 0.0004 |
| 12 | 0 | 0.0079 |
| 13 | 0 | 0 |
| 14 | 0.106 | 0.0022 |
| 15 | 0.015117 | 0.00055 |
| 16 | 0 | 0 |

The UV app data are de-identified, thus only andriod users were able to match by device name, iphone user data were taken average and compared.
