# Supplementary material for: Soft, stretchable, epidermal sensor with integrated electronics and photochemistry for measuring personal UV exposures
Source: PLoS One. 2018 Jan 2;13(1):e0190233. doi: 10.1371/journal.pone.0190233 (PMC5749742; doi:10.1371/journal.pone.0190233)
Supplement: S3 Table — (PDF) [file pone.0190233.s004.pdf]

S3 Table: The individual level data for the clinical study where subjects walked along the pre-set route in the morning, at noon and in the afternoon for four miles, respectively (Unit: MJ/m^2).

	cumulative: cumulative through the day								
	**day 1 walk - morning**			**day 1 walk until noon**			**day 1 walk until afternoon**		
**Subject #**	**Scienterra dosi**	**App reading**	**Patch picture analysis**	**Scienterra dosi**	**App reading**	**Patch picture analysis**	**Scienterra dosim**	**App reading**	**Patch picture analysis**
**1**	0.082902915	0.22594398	0.079	0.170839	0.225944	0.1628	0.204178	0.268047	0.2086
**2**	0.068529911	0.033054516	0.0415	0.139427	NA	0.1296	0.162752	NA	0.1314
**3**	0.073878785	0.2153225	0.05	0.141569	NA	0.1135	0.169095	NA	0.1219
**4**	0.057832077	0.18113472	0.0555	0.110877	0.114989	0.1122	0.1356	0.133941	0.1297
**5**	0.089789178	0.095	0.0969	0.172158	0.163913	0.1666	0.201286	0.163913	0.1828
**6**	0.082880315	0.072133	0.0785	0.153779	0.078829	0.1459	0.179219	0.247234	0.1962
**7**	0.075531628	0.3389	0.0899	0.151196	0.444218	0.1926	0.176525	0.444218	0.2026
**8**	0.074974963	0.06641	0.0689	0.14649	0.1632	0.159	0.175888	0.256409	0.1885
**9**	0.082633124	0.20931	0.102	0.157396	0.20931	0.1798	0.189233	0.20931	0.1963
**10**	0.067419493	0.069626	0.0678	0.136372	0.248126	0.1699	0.163794	0.248126	0.1837

The Scienterra dosimeter readings were readings from Scienterra dosimeter badges. Subjects scanned the patches with the pre-installed smartphone app. At the same time, patch pictures were also taken by a trained instructor. Patch images, UV dosimeter readings and app readings were compared. The app on subject 2 and 3's Android devices did not function for the noon and afternoon walk due to device limitations, therefore data was not received.
